# Radiation-induced maculopathy and fluocinolone acetonide implant

**DOI:** 10.1007/s00417-025-06916-4

**Published:** 2025-08-14

**Authors:** Jules Fricker, Sacha Nahon-Esteve, Sarah Tick, Maxime Nhari, Géraldine Chotard, Anh-Minh Nguyen, Stéphanie Baillif, Laurent Kodjikian, Thibaud Mathis

**Affiliations:** 1 Service d’Ophtalmologie, Hôpital Universitaire de la Croix-Rousse, Hospices Civils de Lyon, 103, Grande Rue de la Croix-Rousse, Lyon, 69317 France; 2 Service d’Ophtalmologie, Hôpital Pasteur 2, Centre Hospitalier Universitaire de Nice, Université Côte d’Azur, Nice, 06000 France; 3 INSERM, Biologie et Pathologies des mélanocytes, équipe 1, Equipe labellisée Ligue 2020 and Equipe labellisée ARC 2019, Centre Méditerranéen de Médecine Moléculaire, Nice, 06200 France; 4 Service Oncopole, Hôpital National de la Vision des 15-20, Paris, 75571 France; 5 Centre Ophtalmologique Pôle Vision Val d’Ouest, Lyon, France; 6 Laboratoire MATEIS, UMR-CNRS 5510, INSA, Université Claude Bernard, Lyon 1, Villeurbanne, 69100 France

**Keywords:** Fluocinolone acetonide implant, Macular edema, Ocular tumor, Radiation-induced maculopathy

## Abstract

**Purpose:**

This study evaluates the effectiveness of fluocinolone acetonide (FAc) implants in managing radiation maculopathy, a complication of ocular irradiation. The primary outcomes were the change in best-corrected visual acuity (BCVA) and central foveal thickness (CFT), and the impact on therapeutic burden and intraocular pressure (IOP).

**Methods:**

This was a retrospective, multicenter, observational study conducted across three French oncology-ophthalmology reference centers. Twelve eyes of twelve patients with radiation maculopathy following ocular irradiation for uveal melanoma were included. All patients received at least one injection of the FAc implant between February 2021 and July 2023. Data on tumor characteristics, lens status, BCVA, CFT, IOP, and SD-OCT findings were collected. The number and types of intravitreal injections before and after FAc implantation were analyzed.

**Results:**

Median final BCVA improved significantly to 72.5 ETDRS letters (IQR 57.5–76.2) (Snellen equivalent: 20/32) (*p* = 0.03). Median final CFT decreased significantly to 328.0 μm (IQR 286.0-405.0) (*p* = 0.04). The therapeutic burden was significantly reduced, with the mean injection interval increasing from 4.2 to 6.5 months (*p* = 0.03) and the annual number of injections decreasing from 3.2 to 0.2 (*p* < 0.001). IOP significantly increased during follow-up, peaking at 21.5 mmHg (IQR 18–25) (*p* < 0.001) but returned to the baseline of 13 mmHg after the treatment adjustments made at the final visit (IQR 11.5–17.2) (*p* = 0.20).

**Conclusion:**

FAc implant improved BCVA and significantly reduced the therapeutic burden in patients with radiation maculopathy. However, careful IOP monitoring is required to effectively manage intraocular hypertension.

**Supplementary Information:**

The online version contains supplementary material available at 10.1007/s00417-025-06916-4.

## Key messages

*****What is known?***:**

- Current treatments for radiation maculopathy are based on the extrapolation of therapeutic strategies used for similar retinal vascular diseases, primarily involving repeat subconjunctival or intravitreal injections of steroid agents, or anti-VEGF agents.
- Intravitreal fluocinolone acetonide (FAc) implants with sustained-release properties have shown promising levels of efficacy for treating diabetic or inflammatory macular edema.

*****What is new?***:**

- FAc implants could be therefore integrated into the therapeutic arsenal for managing radiation maculopathy, potentially reducing the treatment burden for patients.
- FAc implant injections led to a significant improvement in final BCVA, and a significant extension of the interval between injections, thus reducing the therapeutic burden for patients.
- Early management of OHT resulted in no significant difference between baseline and final IOP levels, and no cases of new-onset glaucoma were reported.

**Supplementary Information:**

The online version contains supplementary material available at 10.1007/s00417-025-06916-4.

## Introduction

Radiation maculopathy (RM) is a serious, common, and progressive complication of ocular radiotherapy, which is used as a conservative treatment for ocular tumors [1]. Although this approach preserves the anatomical and functional integrity of the eye [2], RM, often complicated by cystoid macular edema, typically appears 12 to 18 months post treatment [3, 4]. Its incidence varies according to the type of tumor, affecting approximately 40% of patients with uveal melanoma [5]. Patients who suffer from this complication typically describe a gradual deterioration in visual acuity, leading to significant vision loss.

From a pathophysiological perspective, RM shares similarities with other retinal vascular diseases such as diabetic retinopathy or retinal vein occlusion. It is associated with radiation-induced endothelial damage which triggers an inflammatory response and progressive retinal ischemia [6], disrupting the blood-retinal barrier and causing chronic edema. Current treatments for RM, extrapolated from those used for similar retinal vascular diseases [7, 8], mainly involve repeat subconjunctival [9], or intravitreal injections of steroid [10] or anti-VEGF agents [11]. However, these approaches require repeat administration and can lead to ocular complications, including endophthalmitis, traumatic or corticosteroid-induced cataracts, and retinal detachment [12]. Moreover, the therapeutic burden of repeat visits and injections can lead patients to discontinue their treatment, particularly those also undergoing systemic cancer treatment.

Recently, the sustained-release fluocinolone acetonide (FAc) intravitreal implant has been shown to be effective in treating macular edema, particularly of diabetic or inflammatory origin [13]. Using this implant to treat RM could constitute an attractive therapeutic alternative thanks to the reduced frequency of injections and the improved visual acuity and central macular thickness obtained [14].

The objective of this study is to describe a case series of patients treated with the FAc implant for RM.

## Methods

### Patient selection

We conducted a retrospective, observational, multicenter study across three French onco-ophthalmology reference centers in France: Hôpital des Quinze-Vingts (Paris), Hôpital Pasteur 2 (Nice), and Hôpital de la Croix-Rousse (Lyon). All patients who developed RM, complicated by cystoid macular edema, following irradiation for uveal melanoma (60 Gy_RBE dose delivered in 4 fractions of 15 Gy over 4 days) and who received at least one injection of FAc implant were included. All cases were treated with the same FAc implant (Iluvien^®^, Horus Pharma, Nice, France) between February 2021 and July 2023. Patients with a follow-up period of less than six months after the FAc implant injection were excluded. The study protocol was conducted in accordance with the Declaration of Helsinki. Informed consent was obtained from all patients, and the study received approval from a local ethics committee (Hospices Civils de Lyon) under the registration number 20–156.

### Data collection

Epidemiological data were collected retrospectively from patients’ medical records. These included demographic data, medical and ophthalmological history, date of diagnosis, tumor characteristics, lens status, intraocular pressure (IOP), and best-corrected visual acuity (BCVA) measurements using the ETDRS scale. The number and type of intravitreal injections administered before and after the FAc implant injection were also recorded.

Measurements of BCVA, IOP, as well as slit-lamp examination, fundoscopy, and multimodal imaging results, including spectral-domain optical coherence tomography (SD-OCT, Spectralis, Heidelberg Engineering, Heidelberg, Germany), were collected at baseline, prior to FAc implant injection and at each follow-up visit. Analyses included the measurement of central foveal thickness (CFT). CFT was calculated using the SD-OCT software algorithm, based on the thickness of a 1-mm circular zone centered on the fovea, defined as the combined measurement of the central neurosensory retina, including any intraretinal or subretinal fluid. Manual review of the images was conducted to exclude artifacts and segmentation errors.

### Outcome measures

We first assessed the efficacy of the FAc implant by analyzing changes in BCVA and CFT at various follow-up stages: at baseline, prior to the first FAc implant injection and at the final follow-up visit. Next, we evaluated the reduction in therapeutic burden for the patient by examining the number and frequency of intravitreal injections administered before and after the FAc implant injection. Finally, we analyzed complications associated with intravitreal injections of FAc implant, monitoring the occurrence of endophthalmitis, retinal detachment and ocular hypertension (OHT) requiring hypotensive medication, laser trabeculoplasty, or filtering surgery. IOP was measured before and after the FAc implant injection, with an analysis of peak IOP and the value at the last follow-up visit for each patient.

### Statistical analyses

Categorical variables were presented as frequencies and proportions and compared using Chi-square tests. Continuous variables were expressed as medians with interquartile ranges and compared using Wilcoxon matched-paired signed rank tests. Qualitative variables were compared using McNemar’s test. All tests were two-sided, with a p-value < 0.05 considered to be statistically significant. All statistical analyses were performed using R software, version 4.2.3 (R Foundation for Statistical Computing, Vienna, Austria).

## Results

### Patient characteristics

This study included 12 eyes of 12 patients analyzed independently. The cohort comprised 10 men (83.3%) and 2 women with a median age of 62.0 years (IQR 53.5–73.7). All of the 12 eyes studied presented RM following oncologic treatment with proton therapy for uveal melanoma. The median tumor thickness was 5.4 mm (IQR 4.5–6.8) and the median tumor diameter was 12.3 mm (IQR 10.9–14.2). The median distance from the macula was 5.4 mm (IQR 3.3–9.3) and the median distance from the optic nerve was 6.2 mm (IQR 4.2–8.9). The median dose received by the macula was 0 Gy (IQR 0-10.5). Median BCVA at tumor diagnosis, before tumor treatment, was 81.5 ETDRS letters (IQR 75.3–83.5) (Snellen equivalent: 20/25), and median CFT was 387.5 μm (IQR 331.5–497.8).

At the time of the FAc implant injection, the median duration of follow-up form the time of the tumor diagnosis was 66.0 months (IQR 56.3–84.0) and patients had been treated for cystoid macular edema for a median of 25.5 months (IQR 16.5–43.0). Ten eyes (83.3%) were pseudophakic, median BCVA had decreased to 65 ETDRS letters (IQR 47.5–71.2) (Snellen equivalent: 20/50), with a corresponding median CFT of 379.5 μm (IQR 334.2–465.5). Median IOP was 12.5 mmHg (IQR 10.0–18.0), with one patient presenting with glaucoma. Before receiving the FAc implant, six patients were treated with intravitreal anti-VEGF injections (bevacizumab, aflibercept or ranibizumab), followed by a secondary switch to DEX implant. The remaining six patients were initially treated with corticosteroids. All patients had received at least three DEX implant injections before the FAc implant. Prior to the FAc implant injection, patients had received a median of 11.0 intravitreal injections (IQR 6.7–15), including a median of 5.5 Dexamethasone (DEX) implants (IQR 3.8–7.0). The median interval between the last three injections was 4.2 months (IQR 3.1–5.0), with a median of 3.2 injections per year (IQR 3.1–5.3). Eight eyes (67%) required repeat injections at an interval of less than five months, while the injection intervals for 4 eyes (33%) exceeded five months. A detailed description of the patients’ baseline characteristics and all intravitreal injections is provided in Table 1.

**Table 1:**
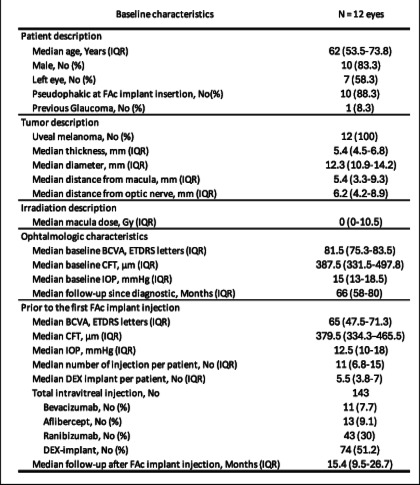
Baseline characteristics of the study population

*No* Number, *IQR* Interquartile range, *FAc implant* Fluorocinolone Acetonide Implant, *BCVA* Best-Corrected Visual Acuity, *ETDRS* Early Treatment Diabetic Retinopathy Study, *CFT* Central Foveolar Thickness, *IOP* Intraocular pressure, *DEX implant* Dexamethasone implant

### Visual acuity and central foveal thickness

**Fig. 1:**
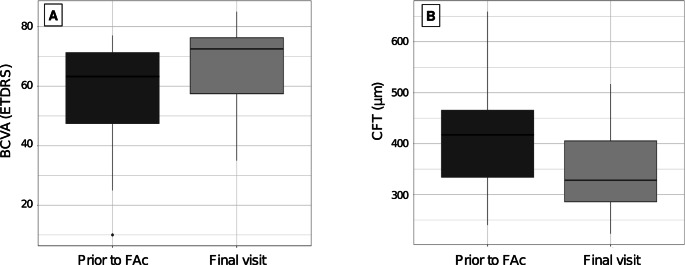
**A**. Comparison between last BCVA before FAc implant and final BCVA (*p* = 0.03). **B**. Comparison between last CFT before FAc implant and final CFT (*p* = 0.04). Box-and-whisker plots represent the interquartile range, with the median value indicated by the line within the box. Whiskers show the range and isolated points represent outliers. FAc implant = Fluorocinolone Acetonide Implant; BCVA = Best-Corrected Visual Acuity; ETDRS = Early Treatment Diabetic Retinopathy Study; CFT = Central Foveolar Thickness

The median follow-up period after the FAc implant injection was 15.4 months (IQR 9.6–25.7). At the final follow-up visit, median BCVA increased significantly to 72.5 ETDRS letters (IQR 57.5–76.2) (Snellen equivalent: 20/32), corresponding to a median BCVA gain of + 7.5 ETDRS letters (IQR 5–10) (*p* = 0.03). Final median CFT decreased significantly to 328.0 μm (IQR 286.0–405.0), corresponding to a reduction of 41.5 μm in CFT (IQR 30.8–97.8) (*p* = 0.04) (Fig. 1).

### Number and frequency of injections

**Fig. 2:**
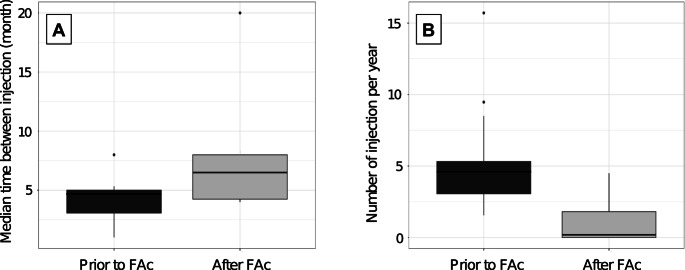
**A**. Median time between injections before and after FAc implant (*p* = 0.03). **B**. Median number of injection per year before and after FAc implant (*p* < 0.001). Box-and-whisker plots represent the interquartile range, with the median value indicated by the line within the box. Whiskers show the range and isolated points represent outliers. IVI = Intravitreal injection; FAc implant = Fluorocinolone Acetonide Implant

Following the initial FAc implant injection, 6 eyes (50%) required an additional intravitreal injection. The median time to the first injection post-FAc implant was 4.0 months (IQR 3.0–4.0). The median number of injections after the FAc implant was 0.5 (IQR 0.0–2.3), with a median interval between injections increasing from 4.2 to 6.5 months (IQR 4.3–8.0) (*p* = 0.03). During follow-up, a total of 18 DEX implants and 1 additional FAc implant were administered for the whole cohort. The median number of injections per year after the FAc implant decreased significantly from 3.2 (IQR 3.0–5.3) to 0.2 (IQR 0.0–1.8) (*p* < 0.001). Two eyes (16.6%) required continued repeat injections at intervals of less than five months, while 10 eyes (83.3%) had injection intervals of more than five months (Fig. 2), demonstrating a significant reduction in injection frequency (*p* = 0.03). A per-eye analysis of the number and frequency of injections is provided in Table 2.

**Table 2:**
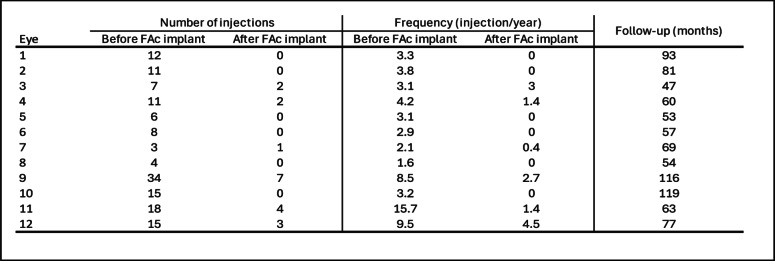
Per-eye count and frequency of intravitreal injections administered before and after the FAc implant, along with the follow-up duration for each patientfa

*FAc implant* Fluorocinolone Acetonide Implant

### Ocular side effects

No cases of endophthalmitis or retinal detachment were reported during the follow-up period. Two of the eyes were phakic at the time of the FAc implant injection and required cataract management during follow-up.

Regarding the incidence of OHT and glaucoma, three patients (25%) experienced mild OHT with IOP ranging between 21 and 25 mmHg, one patient (8.2%) had moderate OHT with IOP between 25 and 30 mmHg, and two patients (16.4%) had high OHT with IOP exceeding 30 mmHg. As a result, therapeutic adjustments were made for six patients (50.0%), with the addition of topical hypotensive medication for all six patients. Additionally, one patient (8.3%) underwent laser trabeculoplasty, and one patient (8.3%) required surgical intervention with trabeculectomy for IOP of 31 mmHg (Table 3).

**Table 3:**
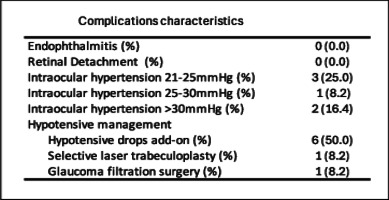
Complications characteristics during follow-up

**Fig. 3:**
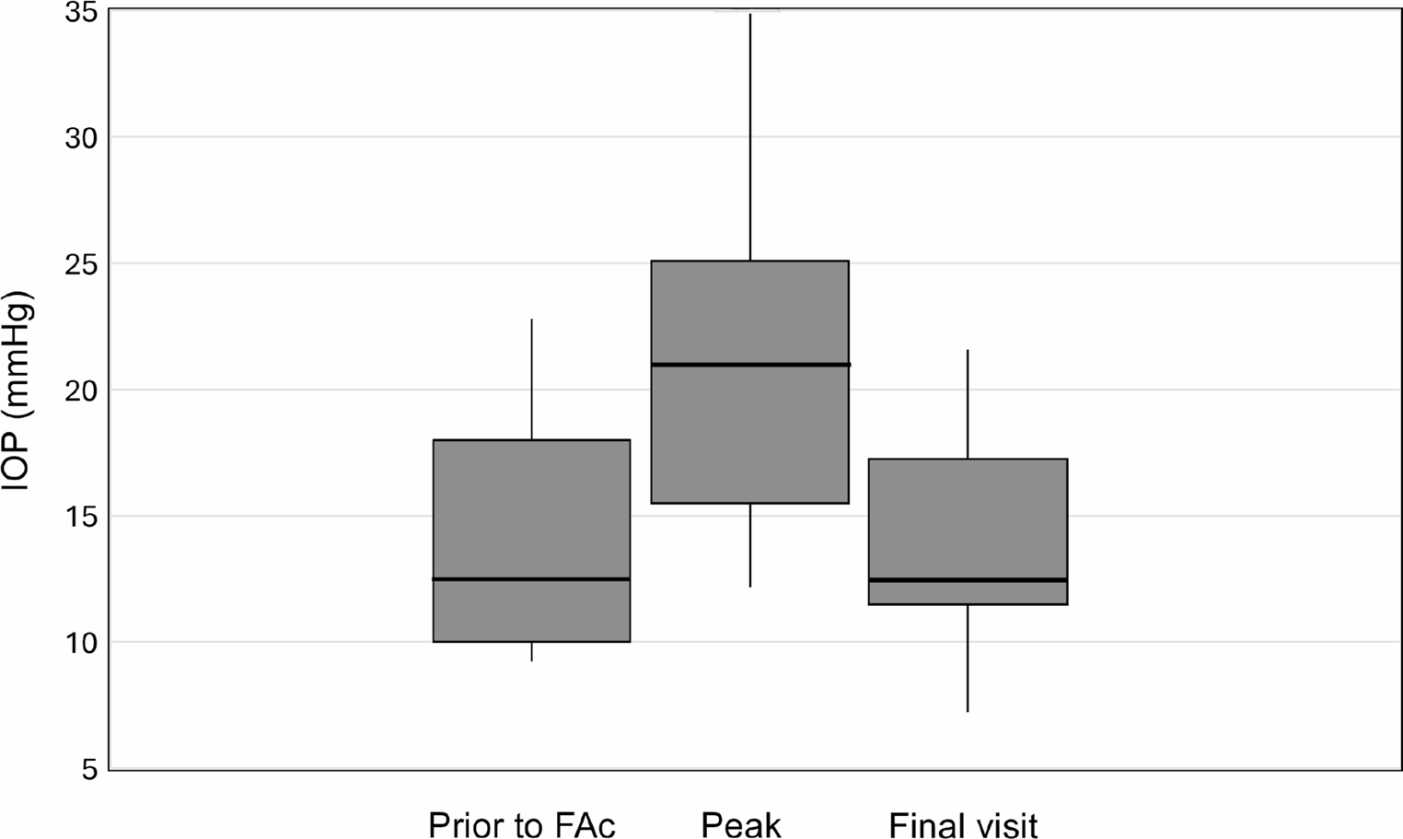
IOP prior to FAc implant injection, at IOP peak and at final visit. IOP increased significantly at IOP peak (*p* < 0.001), but not at final visit (*p* = 0.20). Box-and-whisker plots represent the interquartile range, with the median value indicated by the line within the box. Whiskers show the range and isolated points represent outliers. IOP = intraocular pressure; FAc implant = Fluorocinolone Acetonide Implant

The median IOP peak during the whole follow-up period after FAc injection was 21.5 mmHg (IQR 18.0–25.1), showing a significant increase compared to IOP prior to FAc implant injection (*p* < 0.001). However, final median IOP after treatment for the entire cohort, including patients who developed OHT and required therapeutic modifications, was 13.0 mmHg (IQR 11.5–17.2), which was not significantly different from IOP prior to FAc implant injection (*p* = 0.20) (Fig. 3). No cases of new-onset glaucoma were reported.

## Discussion

This study demonstrates the effectiveness of FAc implant therapy for RM, along with a significant reduction in the therapeutic burden while maintaining a favorable safety profile. The use of FAc implant led to a significant improvement in final BCVA, and a significant reduction in final CFT.

Our efficacy results align with the findings of two smaller case-series evaluating FAc implant in RM [15, 16]. FAc implant therapy aims to prevent macular edema recurrence through sustained steroid release. However, CFT prior to the FAc implant in the patients included showed only mild edema (387.5 μm) as all eyes were already treated with DEX implant and subject to regular follow up. Indeed, numerous reviews and expert consensuses recommend the use of FAc implant within the month following DEX implant injection in order to obtain the rapid resolution of macular edema. This would explain why reduction in CFT may appear clinically low, although statistically significant. Following this injection patients generally benefited from a prolonged period of anatomical stability with no further relapses. In these cases, the aim of the treatment with FAc was to reduce the treatment burden. One example of a patient in this situation is provided in Supplemental Fig. 1. Although BCVA improvement may seem unexpected, studies suggest that stabilizing macular edema fluctuations enhances visual outcomes [17, 18]. Moreover, the ProDEX study studied eyes with macular edema with various etiologies, including RM, treated with DEX implant under a proactive regimen (i.e. reinjection in advance, with a view to preventing macular edema recurrence). The authors’ analyses show that switching from a reactive regimen to a proactive DEX implant regimen significantly improves visual acuity [19]. The present study, and these previous studies emphasize the need for to reduce macular edema fluctuation by improving long-term control of the disease. A therapeutic strategy which incorporates long-standing intravitreal steroid implants, such as DEX implant or FAc implant would help to achieve this aim. The main advantage of FAc implant is the reduced treatment burden compared to anti-VEGF or DEX implant.

Regarding the therapeutic burden, this study demonstrates a significant increase in intervals between injections, resulting in a marked reduction in both annual injection frequency and the proportion of patients requiring injections within five months. These findings align with those reported in the study of the same pathology by Singaravelu et al. [16]. In addition to minimizing procedure-related complications [20], longer injection intervals enhance patients’ quality of life [21], reduce the number of follow-up visits required [22], and the socio-economic costs [23]. However, it should be noted that half of the eyes in this study required another DEX implant injection during follow-up. The FAc implant can be regarded as a prophylactic foundational treatment that prevents macular edema recurrence and subsequent vision loss. Administering a FAc implant decreases the overall recurrence of macular edema, but a DEX implant might still be required in the event of a significant relapse. It is important not to consider this as a failure of the FAc implant, as the overall number of additional treatments and treatment frequency will still be significantly reduced, as described above.

However, the use of sustained-release corticosteroids such as DEX implant or FAc implant requires careful monitoring of IOP and prompt management of ocular hypertension [24]. Prior to considering FAc implant administration, each patient received at least three DEX implants to assess and mitigate the risk of corticosteroid-induced hypertension. Despite these precautions, our study observed a significant increase in maximum IOP following FAc implant injection, with 50% of patients developing OHT (mild to moderate in 66% of cases and severe in 33% of cases). Early management of OHT with additional medical therapy, laser trabeculoplasty, or surgical intervention effectively controlled IOP, resulting in no significant difference between baseline and final IOP levels. Notably, only one patient required filtration surgery, but this patient had pre-existing OHT at the time of RM diagnosis, prior to any intravitreal injections. As described in the drug formulary and supported by long-term studies, IOP elevation observed after FAc implant administration is generally transient. IOP tends to gradually return to baseline levels following the pharmacological washout of the corticosteroid, indicating that the hypertensive effect is reversible once the drug’s activity diminishes [25]. These findings are consistent with the results from princeps studies [26], which reported an OHT rate of 37.1% and a filtration surgery rate of 4.8% [20, 27].

The limitations of our study primarily stem from its retrospective nature, carrying a risk of missing data. In order to limit this risk, we only recruited patients from tertiary oncology and retina centers which are known to collect comprehensive data from oncology patients. Furthermore, the small sample size reflects the rarity of ocular tumors and the limited use of FAc in RM as it is not FDA-approved for this complication at the time this study. Although the aim is to decrease treatment burden and number of injections, the limited number of patients and follow-up do not allow us to draw any conclusions from this cohort. Nonetheless, this study represents the largest cohort to date focusing on the use of FAc implant injections for RM. Furthermore, the median follow-up duration post-FAc implant injection was relatively short, at approximately 14 months, despite the treatment’s efficacy extending up to 36 months. However, it should be noted that the peak effectiveness of the implant is generally reached between 9 and 12 months after injection, which is also when most OHT cases are observed. A longer follow-up period, with repeat measurements of BCVA, CFT and IOP, is essential to thoroughly assess the long-term efficacy and safety of this treatment. Future studies with larger cohorts and an extended follow-up period are required to confirm these findings and better understand the long-term outcomes of FAc therapy in RM.

## Electronic supplementary material

Below is the link to the electronic supplementary material.

Supplemental Figure 1 Example of a patient with radiation maculopathy occurring 13 months after protontherapy for a ciliary body melanoma, treated with DEX implant followed by FAc implant. SD-OCT shows the presence of cystoid macular edema, resulting in a decrease in visual acuity to 77 ETDRS letters. An initial DEX implant was administered, achieving complete efficacy and visual recovery to 85 ETDRS letters at 2 months. However, there was a recurrent pattern of cystoid macular edema every 3 to 4 months, requiring a total of 6 DEX implants to date, while maintaining visual acuity at 85 ETDRS letters. A FAc implant was subsequently administered with DEX implant n°7. No recurrence of cystoid macular edema was observed during the 96 weeks (24 months) of follow-up (though some apoptotic intraretinal cystoid spaces persisted). A slight increase in traction exerted by a secondary epiretinal membrane was also observed. SD-OCT = Spectral Domain-Optical Coherence Tomography; Q = week; DEX implant = Dexamethasone implant; VA = Visual Acuity; IOP = Intraocular Pressure; ETDRS = Early Treatment Diabetic Retinopathy Study; FAc implant = Fluorocinolone Acetonide Implant (PDF 1.28 MB)
