# Supplementary figures and images for: Radiation-induced maculopathy and fluocinolone acetonide implant
Source: Graefes Arch Clin Exp Ophthalmol. 2025 Aug 14;263(11):3219–26. doi: 10.1007/s00417-025-06916-4 (PMC12675579; doi:10.1007/s00417-025-06916-4)

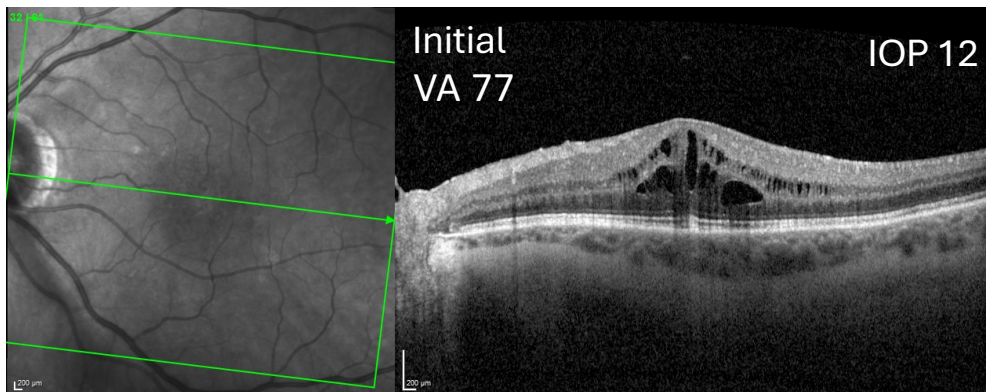

DEX Implant n°1

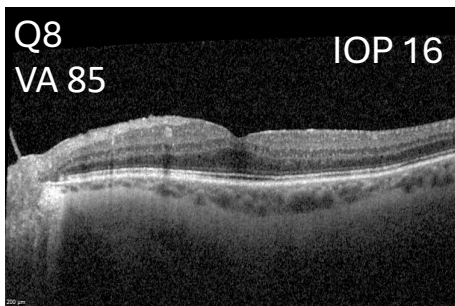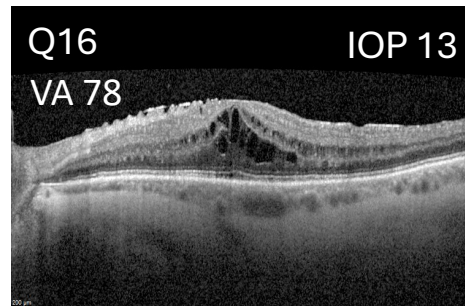

DEX Implant n°6

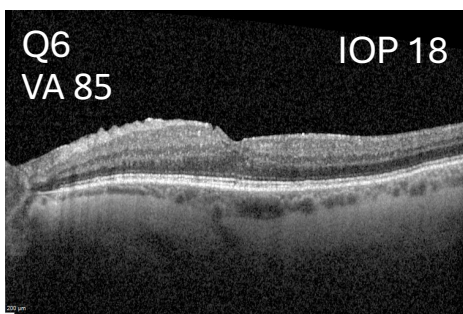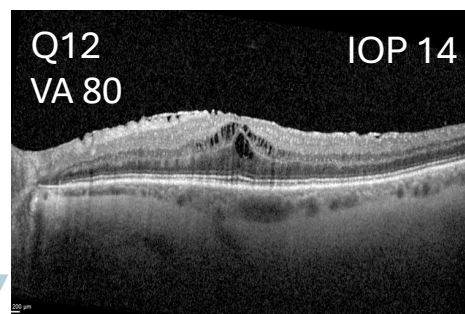

DEX Implant n°7 + FAc Implant

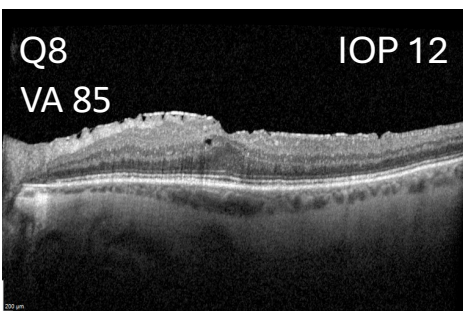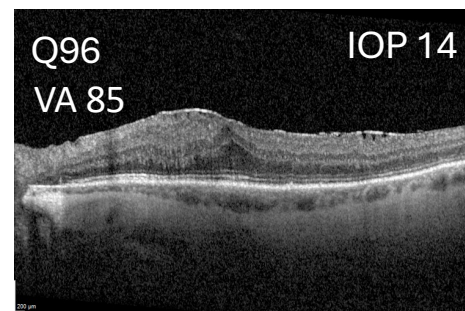

Supplement: Supplementary file 1 — Supplemental Figure 1 Example of a patient with radiation maculopathy occurring 13 months after protontherapy for a ciliary body melanoma, treated with DEX implant followed by FAc implant. SD-OCT shows the presence of cystoid macular edema, resulting in a decrease in visual acuity to 77 ETDRS letters. An initial DEX implant was administered, achieving complete efficacy and visual recovery to 85 ETDRS letters at 2 months. However, there was a recurrent pattern of cystoid macular edema every 3 to 4 months, requiring a total of 6 DEX implants to date, while maintaining visual acuity at 85 ETDRS letters. A FAc implant was subsequently administered with DEX implant n°7. No recurrence of cystoid macular edema was observed during the 96 weeks (24 months) of follow-up (though some apoptotic intraretinal cystoid spaces persisted). A slight increase in traction exerted by a secondary epiretinal membrane was also observed. SD-OCT = Spectral Domain-Optical Coherence Tomography; Q = week; DEX implant = Dexamethasone implant; VA = Visual Acuity; IOP = Intraocular Pressure; ETDRS = Early Treatment Diabetic Retinopathy Study; FAc implant = Fluorocinolone Acetonide Implant (PDF 1.28 MB) [file 417_2025_6916_MOESM1_ESM.pdf]
